# Plasma fatty acids reflect pain, disability, and psychological well-being in knee osteoarthritis in a longitudinal study with joint replacement surgery

**DOI:** 10.1038/s41598-026-36812-8

**Published:** 2026-01-22

**Authors:** Anne-Mari Mustonen, Laura Säisänen, Lauri Karttunen, Amir Esrafilian, Petro Julkunen, Jusa Reijonen, Reijo Käkelä, Sanna P. Sihvo, Juho Kopra, Heikki Kröger, Jari Arokoski, Petteri Nieminen

**Affiliations:** 1 Institute of Biomedicine, School of Medicine, Faculty of Health Sciences, University of Eastern Finland, Kuopio, Finland; 2 Department of Environmental and Biological Sciences, Faculty of Science, Forestry and Technology, University of Eastern Finland, Joensuu, Finland; 3 Department of Clinical Neurophysiology, Kuopio University Hospital, Kuopio, Finland; 4 Department of Technical Physics, Faculty of Science, Forestry and Technology, University of Eastern Finland, Kuopio, Finland; 5 Institute of Clinical Medicine, School of Medicine, Faculty of Health Sciences, University of Eastern Finland, Kuopio, Finland; 6 Neuro Center, Kuopio University Hospital, Kuopio, Finland; 7 Department of Rehabilitation, Kuopio University Hospital, Kuopio, Finland; 8 Department of Bioengineering, Stanford University, Stanford, CA USA; 9 Molecular and Integrative Biosciences Research Programme, Faculty of Biological and Environmental Sciences, University of Helsinki, Helsinki, Finland; 10 Helsinki University Lipidomics Unit (HiLIPID), Helsinki Institute of Life Science (HiLIFE) and Biocenter Finland, Helsinki, Finland; 11 School of Computing, Faculty of Science, Forestry and Technology, University of Eastern Finland, Kuopio, Finland; 12 Department of Orthopaedics, Traumatology and Hand Surgery, Kuopio University Hospital, Kuopio, Finland; 13 Kuopio Musculoskeletal Research Unit, University of Eastern Finland, Kuopio, Finland; 14 Department of Internal Medicine and Rehabilitation, Division of Rehabilitation, Helsinki University Hospital, Helsinki, Finland; 15 University of Helsinki, Helsinki, Finland

**Keywords:** Fatty acid, Functional disability, Knee, Mental health, Osteoarthritis, Pain, Biomarkers, Diseases, Medical research, Pathogenesis, Rheumatology

## Abstract

We investigated the associations of pro- and anti-inflammatory fatty acids (FAs) with cartilage degradation, functional limitations, pain, and psychological well-being in knee osteoarthritis (KOA). Fasting plasma samples were obtained from controls (*n* = 12) and from end-stage KOA patients at baseline (*n* = 13), and 3 months (*n* = 11) and 12 months (*n* = 9) after knee replacement surgery. FA composition in total lipids was analyzed with gas chromatography and mass spectrometry. Cartilage loss was determined by magnetic resonance imaging, and knee pain and disability by physical performance and quantitative sensory testing, neuromuscular examination, and several questionnaires. The associations between variables were tested with the univariate analysis of variance adjusted for age and body mass index. KOA was characterized with elevated baseline 16:1n-7 percentages, while the proportions of 24:0 decreased 12 months after surgery and those of 24:1n-9 decreased 3 and 12 months after surgery. Several FA variables, such as 20:3n-6, 20:4n-6, long-chain saturated FAs, and 24:1n-9, were associated with pain, stiffness, disability, pain self-efficacy, or mental health. Circulating FAs can predict KOA symptoms, independent of age and body adiposity, and provide promising targets to design novel pain treatments.

**Supplementary Information:**

The online version contains supplementary material available at 10.1038/s41598-026-36812-8.

## Introduction

Pain, swelling, stiffness, and impaired joint function are key symptoms of osteoarthritis (OA) – an age-associated degenerative joint disease leading to disability and reduced quality of life^1^. As it is currently impossible to restore damaged cartilage, the treatment goals for OA are to manage pain, slow down disease progression, and postpone joint replacement surgery. The current understanding of the genesis of nociceptive, inflammatory, and neuropathic OA pain is limited^2^. Recommended forms of pain medication, such as non-steroidal anti-inflammatory drugs (NSAIDs) and opioids, often have limited analgesic efficacy but potentially detrimental side effects^1^. OA pain is heterogeneous, varying between individuals and over the course of the disease, and influenced by psychosocial factors^2^. The neurobiological mechanisms of OA pain are complex and inadequately understood, and they involve both peripheral and central sensitization. It is known that knee pain does not necessarily correspond to the radiological findings of OA^3^, and it would be important to assess which biological factors show the strongest correlations to OA pain.

Several biomarkers have been associated with joint degradation or pain severity in patients with OA^1,4^. These include cytokines, adhesion molecules, growth factors, matrix proteins, neoepitopes, and neuropeptides, among others. However, they are not specific to OA pain but are involved in various biological processes, such as tissue growth and turnover, as well as inflammation and pain in general. For instance, tumor necrosis factor-*α* (TNF-*α*), interleukins (IL-6, IL-8), and matrix metalloproteinases have been linked to increased pain severity but, in some studies, TNF-*α*, IL-6, and IL-1*β* have also shown inverse associations with pain. Same markers can reflect specific pain characteristics, such as pain during movement or at rest. Understanding the mechanisms behind nociceptive, inflammatory, and neuropathic OA pain may help unravel the sequence of events leading to and maintaining chronic pain, as well as identify better treatment options for OA patients at different stages of the disease.

Fatty acids (FAs) participate in both physiological and pathological processes and can be measured from body fluids, including plasma and synovial fluid (SF). The involvement of FAs and their lipid mediator (LM) derivatives, such as classic eicosanoids from n-6 polyunsaturated FAs (PUFAs) and specialized pro-resolving mediators (resolvins, protectins, and maresins) from n-3 PUFAs, has been demonstrated in the pathogenesis and pain processes of OA^5^. The identification of specific lipid signatures associated with pain could lead to their use as biomarkers or therapeutic tools for OA. In general, n-3 PUFAs are considered anti-inflammatory, pro-resolving, and anti-nociceptive. There is a loss of n-3 PUFAs in rheumatoid arthritis (RA), and dietary n-3 PUFA supplements can reduce NSAID use by RA patients^6^. They may also decrease painful symptoms in OA patients, but current evidence remains inconclusive^7^. In contrast, n-6 PUFAs and their LM derivatives are generally more pro-inflammatory and potent drivers of inflammation and pain^5^. Regarding knee OA (KOA), plasma n-6/n-3 PUFA ratios showed positive associations with pain symptoms and functional limitations^8^. Dietary n-3 PUFA intake correlated inversely and n-6/n-3 PUFA ratio directly with unacceptable pain and refractory pain in methotrexate-treated patients with early RA^9^. Loef et al.^10^ observed that plasma lipidome was not associated with KOA pain or function, while it accounted for 12% and 6% of the variance in the severity of hand OA pain and functional impairment, respectively. In some other studies, no associations between circulating FAs and knee/hand OA pain were found^11,12^.

Dietary saturated FAs (SFAs), 16:0 and 18:0, increased pain symptoms in trauma-induced OA of rats compared to shorter-chain 12:0 and 14:0, despite no differences in cartilage damage^13^. In a mouse OA model, plasma levels of cholesterol esters CE 18:2, CE 20:4, and CE 22:6 positively correlated with pain behavior^14^. Regarding FA derivatives, Loef et al.^10^ found no associations between plasma LMs, pain, and function in knee or hand OA. In contrast, SF resolvin RvE2 levels were inversely associated with pain scores in patients with different types of arthritis^15^. Administration of RvE1 or RvD1 attenuated inflammatory pain in mouse models without affecting the basal pain perception^16^. Furthermore, 17-hydroxydocosahexaenoic acid and its product, aspirin-triggered RvD1, displayed anti-hyperalgesic properties in rats with complete Freund᾽s adjuvant-induced arthritis^17^. Consequently, LM analogs may have future potential in OA pain relief^5^.

Knowledge about how circulating FA profiles are associated with pain perception and joint function in KOA patients remains limited. We aimed to find novel biological markers that would associate with articular cartilage degradation and both subjective and objective measures of joint pain and function. To achieve this, we utilized magnetic resonance imaging (MRI), several questionnaires, physical performance assessments, quantitative sensory testing, and neuromuscular and -functional evaluation using navigated transcranial magnetic stimulation (nTMS). The determined FA signatures were correlated with a plethora of measures, including cartilage degradation, pain, sensation, and physical performance before and after total knee arthroplasty (TKA). We hypothesized that (*i*) the presence of KOA, as indicated by cartilage loss and both subjective and objective measures of pain and function, would be reflected in inflammation-related plasma FAs, and that (*ii*) temporal alterations in FA composition would be associated with pain symptoms and joint function before and after TKA.

## Materials and methods

### Subjects, ethics, and sampling

The study included 13 patients (4 men, 9 women) with end-stage primary KOA who underwent TKA at Kuopio University Hospital, along with 12 healthy volunteers (6 men, 6 women) with no reported history of joint disorders (Table 1). The control participants were enrolled from hospital staff and university students. The specific inclusion criteria for the KOA patients were: 45–70 years of age, radiographic evidence of moderate to severe KOA (Kellgren–Lawrence grades 2–4), knee joint pain during most days for the last 12 months, relatively normal range of motion (ROM), no clinical instability of the knee, and normal mental status. The exclusion criteria were as follows: severe knee pain caused by other conditions, such as trauma, infections, or autoimmune diseases, limited ROM (severe flexion or extension deficit), or substantial instability requiring, e.g., hinged knee arthroplasty, metabolic or neurological diseases, continuous use of central nervous system agents, active malignancies, inflammatory arthritis, prior knee replacement surgery, metal implants or devices in the body incompatible with MRI or nTMS, cardiac pacemaker, pregnancy, body mass index (BMI) > 33 kg/m^2^, or thigh circumference > 52 cm (measured 12 cm above the lower margin of the patella). Patients who required expedited surgery due to uncontrollable pain or avascular necrosis of the knee were also excluded.

**Table 1:** Baseline characteristics of the controls and patients (mean ± SE).

Group	Control	OA	*p*
Sex (M/F)	6/6	4/9	0.428
Age (years)	32.7 ± 2.70	61.8 ± 1.75	3.7 × 10^–9^
Body mass (kg)	79.7 ± 3.99	91.5 ± 3.02	0.026
BMI (kg/m^2^)	26.0 ± 0.80	32.1 ± 0.55	0.000001
VAS pain (mm)	0.0 ± 0.00	30.8 ± 7.12	0.001
Total stiffness score^a^	nd	4.0 ± 0.49	n/a
Total physical function score^a^	nd	29.8 ± 2.86	n/a
Min medial tibia cartilage thickness (mm)	1.2 ± 0.09	0.5 ± 0.10	0.000024
Min medial femur cartilage thickness (mm)	0.5 ± 0.03	0.3 ± 0.09	0.206

OA, osteoarthritis; M, male; F, female; BMI, body mass index; VAS, visual analog scale; nd, not determined; sex ratios were tested with the Fisherʼs exact test; the other variables with the Studentʼs t-test; ^a^ from the WOMAC questionnaire.

The Ethical Committee of Kuopio University Hospital approved the study protocol (#140/2017, amendment 8/2020) in accordance with the Declaration of Helsinki. All participants gave written informed consent for the donation of their samples. Baseline characteristics of the subjects, including age, gender, body weight, height, and BMI, were collected. After an overnight fast, KOA patients provided venous blood samples prior to TKA (*n* = 13) and at follow-up visits 3 months (*n* = 11) and 12 months (*n* = 9) after the operation. For the control participants (*n* = 12), the samples were collected only at baseline. Blood was collected into BD Vacutainer K2 EDTA tubes (Becton, Dickinson and Company, Franklin Lakes, NJ, USA) and centrifuged at 2500 × *g* for 15 min at room temperature. To ensure complete platelet removal, the upper plasma layer was subjected to a second centrifugation under the same conditions. The resulting platelet-poor plasma was divided into aliquots and stored at − 80 °C. Comparative OA SF samples (*n* = 10) were aspirated from knee joints during TKA using sterile needles and syringes and stored at − 80 °C.

### FA analysis

Platelet-poor plasma and unprocessed SF samples (100 μl for both) were subjected to transmethylation by heating with 1% v/v H_2_SO_4_ in MeOH under a nitrogen atmosphere. The formed FA methyl esters (FAMEs) were extracted into hexane, dried using anhydrous Na_2_SO_4_, and quantified using the Shimadzu GC-2010 Plus gas chromatograph with a flame ionization detector (Shimadzu, Kyoto, Japan)^18^. Automatic peak integrations were manually checked, and any inaccuracies were corrected using the GCMSsolution *v*4.30 software (Shimadzu). Electron impact mass spectrometry, performed by the Shimadzu GCMS-QP2010 Ultra with a mass selective detector, was used to confirm the structures of the FAMEs. The transmethylation reaction also converted alkenyl chains of plasmalogen phospholipids (PLs) into dimethyl acetals (DMAs). FA composition results, including these alkenyl chains, are expressed as mol-% in plasma and SF total lipids. Related sums, indices, and ratios were calculated as described previously^18^.

### Measures of physical medicine, neuromuscular function, and cartilage thickness

The FA results were correlated with data collected during outpatient visits before and after surgery. The procedures for data collection have been described in detail in an earlier study, which included part of the baseline data^19^. The assessments included: a) Self-reported questionnaires (Western Ontario and McMaster Universities Osteoarthritis Index WOMAC^20,21^, visual analog scale VAS^22^, painDETECT score^23^, pain self-efficacy questionnaire PSEQ^24^, RAND-36 measure of health-related quality of life^25^, and indicators of mental health (Beck depression inventory BDI, Beck anxiety inventory BAI^26^)); b) Physical performance measures (ROM of the knee joints^27^, 30-s chair-stand test, 40-m fast-paced walk test, and 12-step stair-climb test^28^); and c) Quantitative sensory testing (pressure pain threshold PPT, thermal detection and heat pain thresholds^29,30^, and two-point discrimination TPD^31^).

PPT was defined as the minimum force applied to a specific area with a hand-held digital pressure algometer that was perceived as painful. PPTs can be reduced in OA, indicating increased pain sensitivity^29^. OA patients may also experience thermal hypoaesthesia, which was assessed by determining the cutaneous detection thresholds for warm, cold, and heat pain^30^. Thermal detection was measured using a thermode placed on the *vastus medialis* muscle with the Medoc Pathway system (Medoc Ltd., Ramat Yishai, Israel). At the start of the test, skin temperature was stabilized at 30 °C. Participants pressed a button when they perceived a change in temperature (toward cold or warm) and when the sensation became painful or distinctly unpleasant, such as stinging or burning. TPD threshold was defined as the minimum distance between caliper points at which the subject could clearly detect two distinct points instead of one^31^. An increase in TPD threshold indicates reduced tactile acuity, which can occur in OA.

The resting motor threshold (rMT), motor mapping of the *tibialis anterior* muscle, and long-interval cortical inhibition (LICI) were assessed using nTMS following previously described procedures^19^. The LICI likelihood was determined as the % of fully inhibited motor evoked potentials across 20 trials. For these neurophysiological outcomes, the value representing the KOA side was derived from the opposite cerebral hemisphere. Physical performance measures, VAS scores, PPT, and TPD were not collected at 3 months after surgery. Additionally, questionnaire data were not collected from the controls, with the exception of VAS. Due to patient drop-out, complete datasets were not available for all individuals. FA data were also compared to cartilage thickness measurements extracted from the MRI scans of control and OA knees at baseline^19^. The regions of interest were located in the middle and adjacent to the principal load-bearing areas within the medial and lateral compartments of the tibia and femur. As the lateral side showed no substantial KOA-related alterations, it was excluded from subsequent analyses.

### Statistical analyses

All statistical analyses were conducted using the IBM SPSS *v*27 software (IBM, Armonk, NY, USA), except for the correlation plots, for which R *v*4.3.1^32^ with the corrplot package^33^ was utilized. A *p*-value below 0.05 was considered indicative of statistical significance, and the data are reported as the mean ± standard error of the mean (SE). Group and timepoint differences were assessed using the generalized linear model (GLM) and the supervised linear discriminant analysis (DA). Spearman correlations (r_s_) were computed for all variable pairs across combined timepoints, with plasma and SF analyzed separately. To further explore key relationships between FA proportions and variables of cartilage loss, physical disability, pain, and neuromuscular function, the univariate analysis of variance (ANOVA) adjusted for the confounding factors of age and BMI was performed.

## Results

### General variables

The average ages, body masses, and BMIs were higher in the KOA group compared to the controls (Table 1). TKA did not induce significant weight loss (BMI: –0.7 ± 0.94% at 3 months, + 1.9 ± 0.86% at 12 months compared to baseline).

### Effects of KOA and TKA on FA proportions

**Fig. 1:**
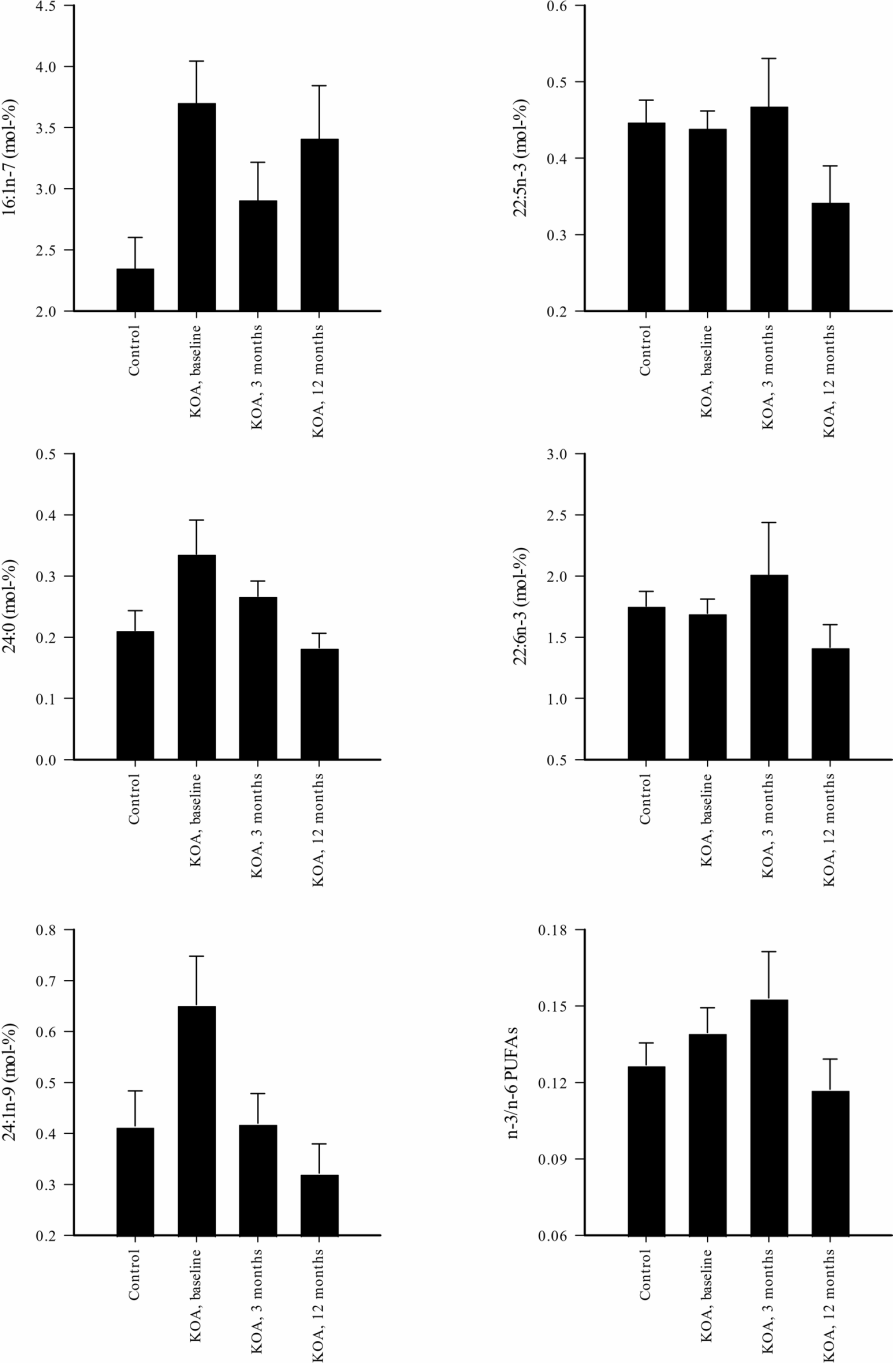
Effects of knee osteoarthritis (KOA) and replacement surgery on selected fatty acid (FA) proportions in plasma (mean + SE). The mol-% proportions were determined at baseline and at 3 months and 12 months post-surgery. PUFA = polyunsaturated FA. All FAs show statistically significant differences between study groups (generalized linear model, *p* < 0.05).

We first assessed the differences in FA composition between the study groups (control, KOA baseline, and KOA 3 and 12 months after TKA). KOA was characterized with elevated baseline 16:1n-7 percentages in plasma, while the proportions of 24:0 were lower 12 months after TKA and those of 24:1n-9 reduced 3 and 12 months after TKA (GLM, *p* = 0.016–0.042; Fig. 1, Supplementary Table S1). Three out of the 57 measured FAs (16:1n-5, 20:0, and 24:1n-9) showed significant negative correlations (r_s_ = –0.762 to –0.881, *p* = 0.004–0.028) in mol-% between the plasma and SF of KOA patients at baseline; none showed positive correlations. Therefore, plasma and SF FA profiles did not appear to be reliable proxies for one another.

The supervised DA classified 98.2% of the samples into their correct study group/timepoint. One KOA plasma sample at 12 months after surgery was misclassified among KOA plasma samples at 3 months after surgery. The most important FA contributors to the model were 18:2n-6, 14:1n-5, 18:0*i*, 17:0*ai*, 12:0, 17:1n-8, and 20:4n-6 in order of relevance. The discriminant function 1 (on the horizontal axis of Supplementary Fig. S1) explained 63.8% of the variance in the dataset and separated KOA SFs from plasma samples. Especially control plasma samples were separated from KOA plasma samples at 3 months after TKA by the discriminant function 2 (on the vertical axis of Supplementary Fig. S1) explaining 22.5% of the variance.

### Associations of FA profiles and KOA symptomatology

**Fig. 2:**
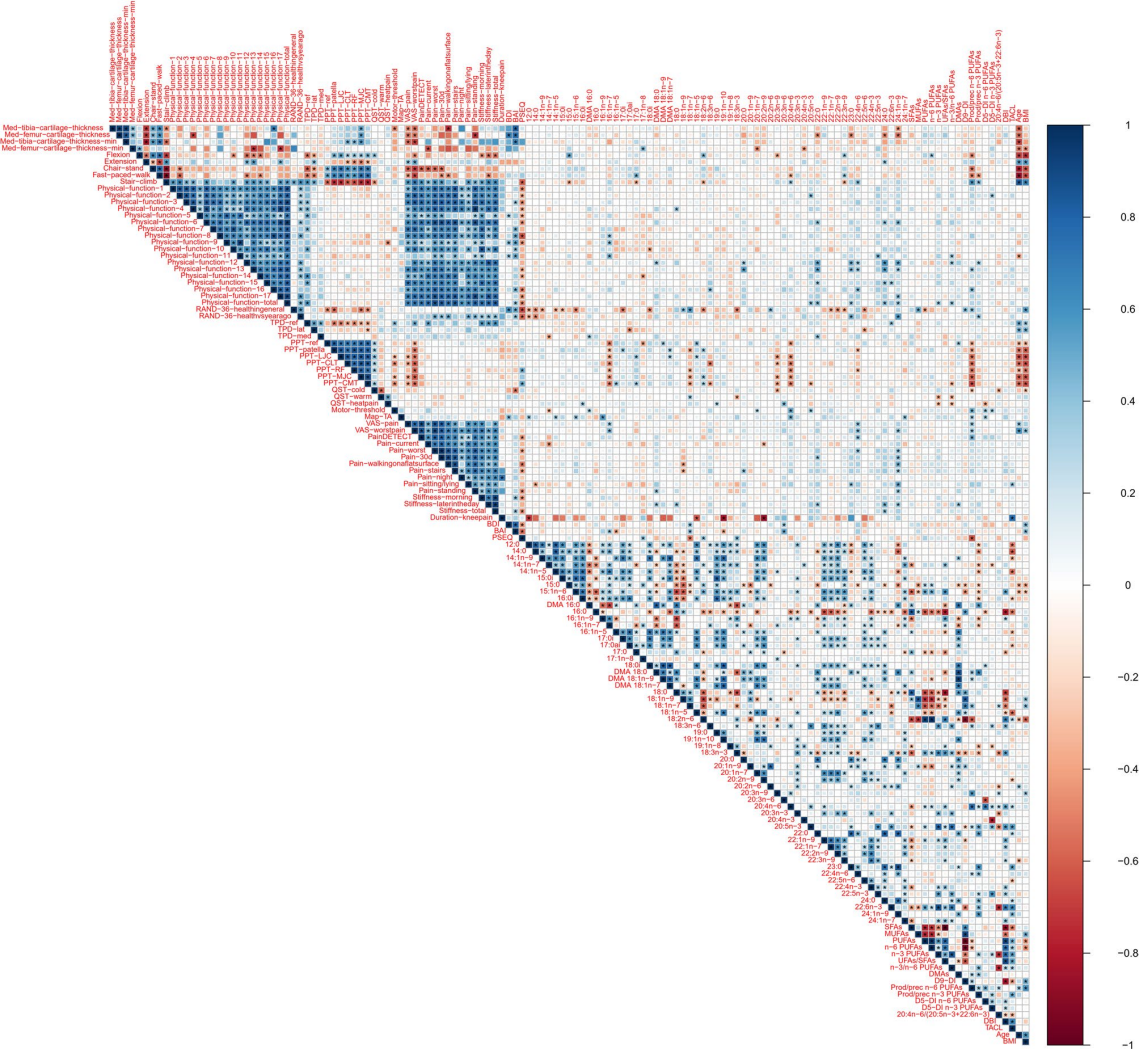
Correlogram showing the Spearman correlation coefficients between plasma fatty acids (mol-%, in the order of chromatographic retention time) and other measured variables. Correlations were computed across all control and osteoarthritis patients and categorized according to the variable groupings. Correlation coefficients are color-coded as indicated. Dark blue (respectively red) on the correlogram indicates strong positive (respectively negative) correlations. Asterisks indicate statistically significant correlations (*p* < 0.05). Definitions of the physical function categories are as follows: 1 = ascending stairs, 2 = descending stairs, 3 = getting up from a sitting position, 4 = standing, 5 = bending down, 6 = walking on a flat surface, 7 = getting in/out of the car, 8 = shopping, 9 = putting on socks, 10 = rising from bed, 11 = taking off socks, 12 = lying in bed (changing or adjusting position), 13 = bathing (bath/shower/sauna), 14 = sitting, 15 = getting on/off the toilet, 16 = heavy domestic duties, 17 = light domestic duties.

Next, we analyzed how FA markers associated with subjectively and objectively assessed joint pain and function by using a systematic analysis of pairwise Spearman correlations between all variables, across the controls and KOA patients, but separately for plasma and SF. Numerous statistically significant correlations were observed (Fig. 2, Supplementary Fig. S2). With the univariate ANOVAs, we further tested the most interesting of these associations with adjustment for potential confounders, age and BMI. The significant findings are presented in Table 2 (plasma FAs) and Supplementary Table S2 (SF FAs).

**Table 2:** Univariate analyses of variance for physiatric and neuromuscular measurements explained by different fatty acid variables in the plasma of controls and knee osteoarthritis patients. Only analyses that are statistically significant when adjusted for age and body mass index are reported.

Fatty acid	Dependent variable	R squared	F	*P*
14:0	Health compared to one year ago (RAND-36)	0.257	6.932	0.014
16:1n-7	Pressure pain threshold, med. tibial condyle	0.498	4.741	0.038
18:1n-9	Pain, walking on a flat surface	0.173	4.766	0.039
18:1n-7	Threshold for warm detection	0.220	5.462	0.025
18:3n-3	Pain self-efficacy	0.288	5.784	0.024
20:3n-6	Threshold for heat pain	0.193	6.723	0.013
	Beck anxiety inventory	0.481	15.164	0.001
20:4n-6	Pressure pain threshold, med. tibial condyle	0.544	8.120	0.008
	Resting motor threshold	0.268	10.043	0.003
	Map *tibialis anterior*	0.235	4.868	0.035
20:5n-3	Resting motor threshold	0.234	7.825	0.008
20:0	Pain, stairs	0.151	4.349	0.047
22:0	Stiffness, later in the day	0.188	5.120	0.032
	Physical function, total	0.186	4.973	0.035
23:0	Physical function, total	0.179	4.748	0.039
	Health compared to one year ago (RAND-36)	0.316	9.661	0.005
24:0	Extension	0.467	7.039	0.013
	Stiffness, later in the day	0.191	5.222	0.031
	Stiffness, total	0.154	4.283	0.049
24:1n-9	Pain, worst	0.201	6.060	0.021
	Pain, 30d	0.236	6.738	0.015
	Pain, walking on a flat surface	0.330	11.762	0.002
	Pain, stairs	0.241	7.814	0.010
	Pain, sitting or lying down	0.229	5.579	0.026
	Stiffness, later in the day	0.199	5.537	0.026
	Physical function, total	0.222	6.315	0.019
	Health compared to one year ago (RAND-36)	0.374	12.883	0.001
ARA/(EPA + DHA)	Threshold for cold detection	0.303	8.629	0.005
Prod/prec (n-6)	Pressure pain threshold, *rectus femoris*	0.350	5.107	0.032
	PainDETECT score	0.241	7.115	0.013
Delta5-DI (n-6)	Resting motor threshold	0.199	5.766	0.021
	Map *tibialis anterior*	0.229	4.613	0.040
TACL	Duration of knee pain	0.470	5.601	0.050

ARA, 20:4n-6; EPA, 20:5n-3; DHA, 22:6n-3; Prod/prec (n-6), (20:3n-6 + 20:4n-6)/18:2n-6; Delta5-DI (n-6), 20:4n-6/20:3n-6; TACL, total average chain length; med., medial.

**Fig. 3:**
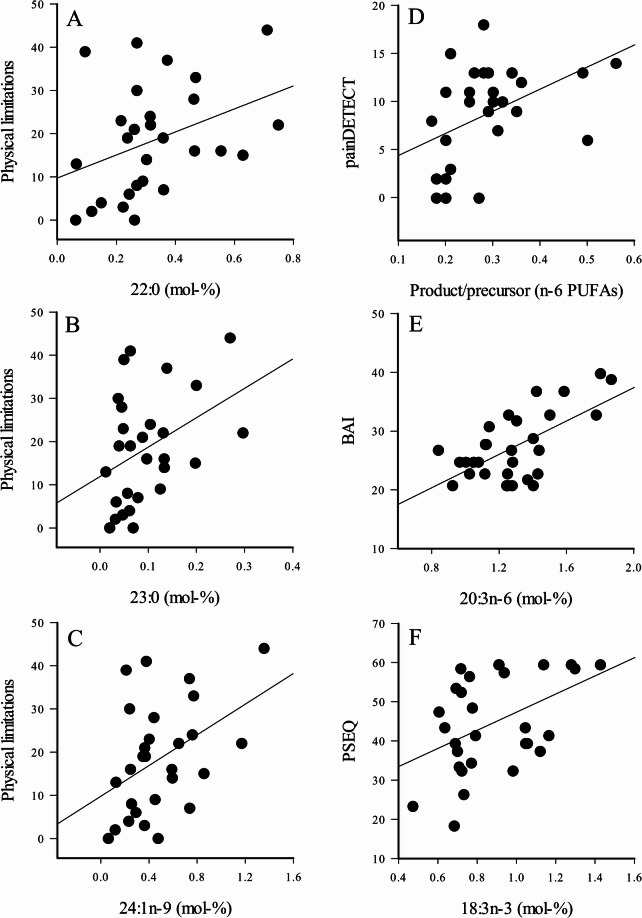
Scatter plots depicting the interrelationships between selected plasma fatty acid variables and physical limitations (A–C), painDETECT scores (D), Beck anxiety inventory (BAI) scores (E), and pain self-efficacy (PSEQ) scores (F) in osteoarthritis patients. The R^2^ and *p* values can be found in Table 2. PUFA = polyunsaturated fatty acid.

Regarding plasma, 20:4n-6, 20:5n-3, and ∆5-desaturation index (DI) of n-6 PUFAs were associated with rMT (Supplementary Fig. S3A–B), and 20:4n-6/(20:5n-3 + 22:6n-3) ratio, 18:1n-7, and 20:3n-6 with thermal detection thresholds (Table 2). Several FAs, such as 16:1n-7, 18:1n-9, 20:0, 20:4n-6, 24:1n-9, and product/precursor ratio of n-6 PUFAs, were associated with different pain categories, and long-chain SFAs and 24:1n-9 with stiffness and functional limitations (Fig. 3A–D, Supplementary Fig. S3C–D). 14:0, 23:0, and 24:1n-9 were associated with RAND-36 health scores compared to the situation one year ago. In addition, 20:3n-6 was associated with BAI and showed borderline significance with BDI (*p* = 0.058), while 18:3n-3 was associated with PSEQ scores (Fig. 3E–F).

With respect to SF, 16:0, 18:3n-6, 18:3n-3, 22:6n-3, total PUFAs, total alkenyl chains (DMAs), and double bond index were associated with different categories of pain (Supplementary Table S2). 16:0, 18:3n-3, and ∆5-DI of n-6 PUFAs were associated with TPD in the lateral knee or the reference upper extremity. Moreover, ∆5-DI of n-6 PUFAs was associated with BDI and BAI, ∆5-DI of n-3 PUFAs with BDI (Supplementary Fig. S4), and 16:1n-7 with PSEQ. There were no significant associations between plasma/SF FA percentages and medial tibia or femur articular cartilage thicknesses, when adjusted for age and BMI.

## Discussion

**Fig. 4:**
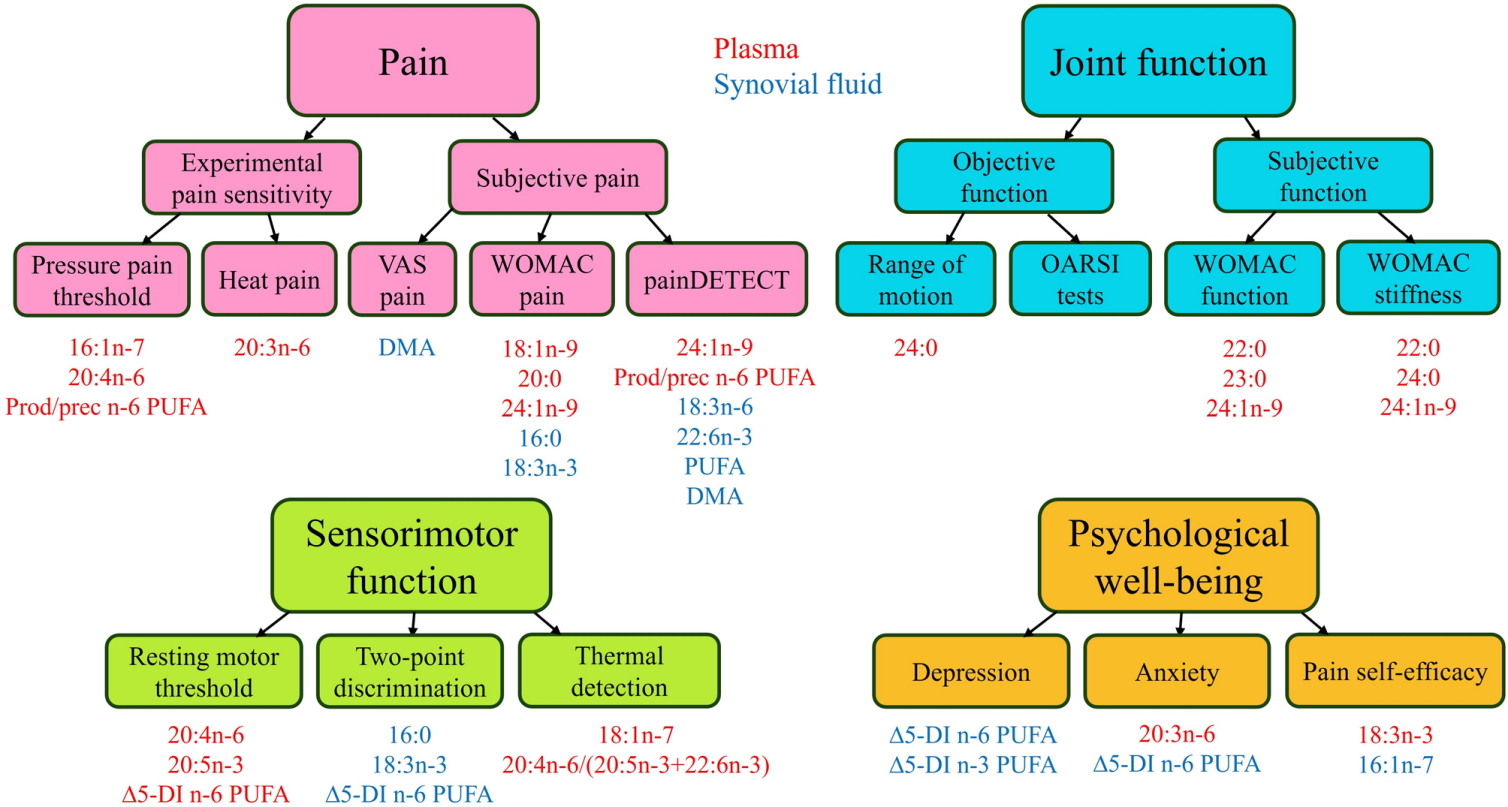
A visual summary of fatty acid (FA) predictors for pain, physical function, sensorimotor function, and psychological well-being. Individual FAs, sums, indices, and ratios that best explained the variability in the measured parameters based on the univariate analysis of variance (*p* < 0.05) are presented with red (determined from plasma) or blue (determined from synovial fluid) font colour. ∆5-DI = delta5-desaturation index, DMA = dimethyl acetal, OARSI = Osteoarthritis Research Society International, Prod/prec = product/precursor, PUFA = polyunsaturated FA, VAS = visual analog scale, WOMAC = Western Ontario and McMaster Universities Osteoarthritis Index.

The present study on KOA correlated longitudinal information from plasma FA profiles to subjective and objective assessments of pain, sensation, physical performance, neuromuscular function, and psychological well-being. Although the percentages of major FAs did not exhibit clear differences between the study groups/timepoints, several FAs were statistically significant predictors of pain, physical limitations and, consequently, quality of life (Fig. 4). The main findings of the study can be summarized as follows: (*i*) n-6 PUFAs, especially 20:4n-6, can increase the risk of chronic knee pain, (*ii*) very-long-chain SFAs and 24:1n-9, abundant in nervous tissue, are associated with knee function, stiffness, and pain in an aggravating manner, (*iii*) 20:5n-3 and 20:4n-6 can play roles in the regulation of corticospinal excitability, and (*iv*) high 20:3n-6 might be connected to psychological distress. These findings suggest that several individual FAs could be involved in pain regulation and functional disability in KOA.

The differences in the average FA percentages between the study groups/timepoints were relatively small and mostly observed in FAs with minor proportions. Baseline 16:1n-7 was elevated by KOA compared to control plasma, which supports earlier data from human SF with higher 16:1n-7 levels in late-stage than in early KOA^34^, and increased 16:1n-7 levels in the cancellous bone of OA patients’ femoral heads^35^. TKA decreased the plasma percentages of 24:0 and 24:1n-9 when measured 3 and/or 12 months after the operation. KOA SF has been previously characterized with elevated concentrations of these very-long-chain FAs^36^, possibly associated with peroxisomal dysfunction, which could prevent the shortening of FAs due to the hindering of their partial *β*-oxidation^37^. On the other hand, 16:1n-7 in serum and SF and 24:1n-9 in serum have previously correlated inversely with OA severity and synovitis, respectively, in a mouse OA model^38^, which makes the interpretation of the data more complicated.

Several plasma FAs showed significant positive (18:2n-6) or negative correlations (18:1n-7, 18:3n-6) with objectively assessed PPTs. In the univariate ANOVAs adjusted for age and BMI, we observed significantly lower PPTs with higher plasma 16:1n-7, 20:4n-6, and product/precursor ratios of n-6 PUFAs. This indicates that, at higher levels of these FA parameters, less pressure would be required to induce pain. The plasma product/precursor ratios of n-6 PUFAs also associated positively and the percentages of 18:1n-9 inversely with self-reported (subjective) pain, while previous studies connected high 18:2n-6, 20:4n-6^39^, and n-6/n-3 PUFA ratios^8^ to greater experimental pain sensitivity. The elongation product of 20:4n-6, 22:4n-6, was noted to negatively correlate with PPTs and to positively correlate with VAS pain and painDETECT scores, which could warrant further studies on the connections of this long-chain n-6 PUFA to the neuropathic component of pain, as well^40^. The findings on 16:1n-7 are also interesting as this monounsaturated FA (MUFA) was previously connected to increased IL-6 secretion by synovial fibroblasts and chondrocytes^41^, and its plasma levels inversely correlated with PPTs and positively with the current and worst VAS pain scores in the present study. We also documented a positive association between pain and SF DMAs, produced by transmethylation of alkenyl chains from plasmalogen PLs, supporting the previously found increase in SF plasmalogen phosphatidylcholine in patients suffering from different painful joint diseases^42^. Plasmalogens, together with sphingomyelin, are among the main PLs of the myelin sheath^43^ and, thus, the association of plasmalogen-derived DMAs with pain could reflect the number and integrity of neural structures exposed to SF in the joint space.

The plasma proportions of C20–24 SFAs and 24:1n-9 showed positive associations with different pain categories, joint stiffness, and physical disabilities. A role for 24:1n-9 in pain perception would not be surprising, as this MUFA is a critical component of sphingolipids in the white matter of the brain and myelinated nerve fibers^44,45^. 24:1n-9 sphingolipids are essential for membrane structure and play important roles in brain development, signal transduction, and neurotransmission, and they can also possess anti-inflammatory properties. In addition, 24:1n-9 shows abnormal levels in recurrent depression, psychosis, and demyelinating diseases. Very-long-chain SFAs are also important constituents of nervous tissue lipids, including sphingomyelins, cerebrosides, and sulfatides^46^. We observed inverse correlations between very-long-chain SFAs, 24:1n-9, and femoral and tibial cartilage thicknesses, but these data did not reach statistical significance in the univariate ANOVAs adjusted for age and BMI. Generally, while there exists evidence on chondroprotective effects of n-3 PUFAs and OA-promoting effects of n-6 PUFAs and SFAs, previous studies have mostly concentrated on a narrow selection of FAs^5^. Notably, data on very-long-chain SFAs and MUFAs are scarce, and the present results suggest that they should be taken into closer scrutiny regarding cartilage health, joint pain, and function.

There were several significant correlations between plasma FA proportions and objectively assessed physical function. For instance, high 18:2n-6 percentages and n-6 PUFA sums were connected to a wider ROM of the knee joint (flexion), and 20:4n-6 and product/precursor ratios of n-6 and n-3 PUFAs to poorer performance in the fast-paced walk and stair-climb tests, but these associations did not remain significant in the univariate ANOVAs adjusted for age and BMI. Our findings are dissimilar to the results of Sibille et al.^8^ on adults with knee pain, in which a high plasma n-6/n-3 PUFA ratio was associated with greater functional limitations compared with the low ratio group. In addition, Loef et al.^10^ documented that lower 18:2n-6 and higher 20:4n-6 levels were associated with greater functional impairment regarding hand but not KOA. Previously, fish oil rich in 20:5n-3 and 22:6n-3 improved WOMAC function scores in KOA patients^47^, but the present study did not demonstrate significant links between n-3 PUFAs and functional categories in the univariate ANOVAs, either.

Plasma 18:1n-7, 20:3n-6, and 20:4n-6/(20:5n-3 + 22:6n-3) ratios were associated with thermal detection, but the significance of these findings remains unclear. Earlier data on the subject are limited, even though dietary 18:3n-6 supplementation was previously linked to improved temperature perception thresholds in patients with mild diabetic neuropathy^48^, while long-chain n-3 PUFAs were without effect in non-alcoholic fatty liver disease patients, both with and without type 2 diabetes^49^. The observed positive association between rMT and 20:5n-3, 20:4n-6, and ∆5-DI of n-6 PUFAs suggests that these FAs could play roles in corticospinal excitability^50^. Among them, 20:4n-6 is a common PUFA in nervous tissue lipids^46^, while brain 20:5n-3 concentrations are typically very low^51^. However, PUFAs and their derivatives (LMs and endocannabinoids) can modulate synaptic function and neurotransmission^52^ and circulating PUFA levels could have value as future biomarkers, as their synthesis rates within the brain are presumably low and, thus, the brain relies on their constant supply from the blood^51^. We also documented inverse correlations between the LICI likelihood and plasma 20:3n-6 and total MUFA percentages, which could hypothetically indicate impaired *γ*-aminobutyric acid type B receptor-mediated intracortical inhibition with high proportions of these FAs^53^. However, these associations did not remain significant in the univariate ANOVAs.

Anxiety scores and pain sensitivity can be highly interrelated in KOA patients, and anxiety at baseline can predict knee pain 12 months later^54^. There was a positive association between plasma 20:3n-6 percentages and anxiety scores in the present study that, to the best of our knowledge, has not been previously documented, and the connection between plasma 20:3n-6 and depression was of borderline significance. The univariate ANOVA explained 48.1% of the variance in BAI and, based on the type III sum of squares, 20:3n-6 emerged as the only significant explanatory factor in this model. Even after adjusting for age, BMI, and VAS pain scores, plasma 20:3n-6 showed positive associations with anxiety (and depression), which clearly merits further studies in the future. Circulating 20:3n-6 has also been previously linked to depression, but earlier studies have yielded mixed results regarding the direction of this connection^55^. The observed positive correlations between plasma 18:1n-7 and depression/anxiety did not remain significant in the univariate ANOVAs with adjustments for age and BMI. On the other hand, long-chain n-3 PUFAs (20:5n-3, 22:6n-3) can have beneficial effects on depression and possibly anxiety^56^, and we also observed an inverse connection between ∆5-DIs of n-3 PUFAs and depression scores, suggesting that, in SF, increased desaturation of 20:4n-3 to 20:5n-3 could be linked to less depressive symptoms. Elevated desaturation of 20:3n-6 to 20:4n-6 in SF was also associated with lower scores of depression/anxiety, which supports our findings on plasma 20:3n-6 as anxiogenic. Moreover, the percentages of 18:3n-3 (plasma) and 16:1n-7 (SF) were positively associated with PSEQ scores, representing the confidence of KOA patients in their ability to perform a particular behavior or activity despite the pain^24^. It has been established that depression is one of the most common comorbidities of OA with recommendations that the depressive symptoms of OA patients, especially as they tend to associate with pain and disability, should be carefully assessed by physicians^57^. Our study offers a new starting point to investigate this comorbidity to help counteract the vicious circle of pain and dysfunction leading to further depression, which can subsequently restrict the rehabilitation of KOA patients even more.

The associations between FA proportions and KOA symptoms were dissimilar in plasma and SF, and the SF FA profiles did not directly follow the plasma FA levels even though SF is considered an ultrafiltrate of plasma^58^. This apparent discrepancy may derive from several factors, such as the release of FAs from degraded joint tissues, increased permeability of the inflamed synovial membrane, altered lymphatic clearance of macromolecules, and elevated levels of inflammatory cells in KOA SF. Particular SF FAs and FA indices showed significance in correlations and univariate ANOVAs with depression and anxiety scores and certain neurological findings, such as TPD. As these FA values were measured from the joint space, their actual significance with skin perception and central nervous processing is not necessarily causal. The turnover rate of molecules can be significantly slower in SF than in circulation^59^, and it could be presumed that the local FA composition of the skin would have more influence on the sensory systems and pain pathways related to this anatomical region. However, the pain-related associations merit further consideration, as OA pain can be associated with the composition of SF^4^. Here, the correlations between pain and generally anti-inflammatory n-3 PUFAs may appear paradoxical and require both prudent interpretation and further research. The beneficial potential of n-3 PUFA supplements in OA pain and joint function has been established^60^, which raises the question of the mechanisms underlying the observed relations between particular n-3 PUFAs and pain perception in the study population. In this case, presumptive explanations include endogenous processes that attempt to counteract joint inflammation and degeneration with resolving metabolites, and nutritional factors, such as the use of dietary PUFA supplements, that may differ in KOA patients *vs.* controls.

There are some limitations to be acknowledged in the present study. The FAs were measured from plasma lipids, where their levels may not reflect the long-term FA intake as accurately as the red blood cell FA signature would^61^. We used mol-% as the measurement unit for FAs because the relative numbers and specific ratios of precursor FAs, rather than their absolute mass-based concentrations in tissues, largely exceeding the need, determine their use for LM synthesis. Furthermore, we were not able to determine FA-derived LMs, and neither was a food frequency questionnaire conducted to assess the dietary FA intake or the use of PUFA supplements. In addition, SF could not be harvested from the controls due to the very limited volume of SF in the healthy knee joint that could not be visualized even with careful ultrasound examination. On the other hand, some previous studies have taken samples at only one timepoint and determined particular PUFAs or FA sums, while the strength of the present experiment was in the measurement of a comprehensive set of individual FAs both before and after TKA.

The small sample size in the present study should also be acknowledged. An *a posteriori* power analysis implied a possibility of type II errors, meaning that significant differences might have been missed. Given this, non-significant results should be interpreted with caution, and replication with a larger sample size is recommended in future studies, while the results with statistical significance remain robust. Another important consideration when interpreting the results is the baseline difference in age and body composition between the control group and the KOA patients. Although efforts were made to match for these confounding factors, it is very difficult in practice to find sufficiently elderly individuals without health issues or any articular symptoms. As a result, the study groups differ in these respects, a limitation that is commonly reported in OA research.

To sum up the present findings, plasma 20:4n-6, 24:1n-9, and product/precursor ratio of n-6 PUFAs were most strongly connected to pain, very-long-chain SFAs and 24:1n-9 to physical limitations and knee stiffness, and 20:3n-6 to anxiety. Many of the observed associations indicate that FAs could play significant roles in inflammatory processes, both pro-inflammatory and, in the case of n-3 PUFAs, potentially pro-resolving, emphasizing the complex nature of FAs in inflammation. The results suggest that circulating FAs may have value as predictors of KOA symptoms, independent of age and body adiposity, and that they could provide promising targets as starting points to design novel pain treatments. While the present study is not sufficient to offer novel dietetic recommendations for KOA patients, the approach of strengthening endogenous resolving processes could be the most promising strategy for preventing the aggravation of cartilage damage in KOA, particularly if applied during the early stages of the disease. Population studies examining FA intake and profiles in KOA patients, correlated with symptomatology, would be crucial to uncover the potential benefits of manipulating FA intake for the treatment of joint disease symptoms.

## Authorsʼ contributions

PN, PJ, HK, and JA designed and coordinated the study. HK collected the samples, and LS, JR, and PJ conducted the physical analyses and LK the physiatric measurements. JR performed the magnetic resonance imaging, and AE determined the thickness of articular cartilage. RK and SPS performed the fatty acid analyses. PN integrated the chromatograms, and PN, A-MM, and JK conducted the statistical analyses and prepared the images. A-MM drafted the manuscript. All authors revised the draft critically and read and approved the final submitted manuscript.

## Supplementary Information

Below is the link to the electronic supplementary material.

Supplementary Material 1

Supplementary Material 2

Supplementary Material 3

Supplementary Material 4

Supplementary Material 5

Supplementary Material 6

## Data Availability

All relevant data analyzed during this study are included in this published article and its supplementary information files.

## Abbreviations

ANOVA: Analysis of variance
BAI: Beck anxiety inventory
BDI: Beck depression inventory
BMI: Body mass index
CE: Cholesterol ester
DA: Discriminant analysis
DI: Desaturation index
DMA: Dimethyl acetal
FA: Fatty acid
FAME: Fatty acid methyl ester
GLM: Generalized linear model
IL: Interleukin
KOA: Knee osteoarthritis
LICI: Long-interval cortical inhibition
LM: Lipid mediator
MRI: Magnetic resonance imaging
MUFA: Monounsaturated fatty acid
NSAID: Non-steroidal anti-inflammatory drug
nTMS: Navigated transcranial magnetic stimulation
OA: Osteoarthritis
PL: Phospholipid
PPT: Pressure pain threshold
PSEQ: Pain self-efficacy questionnaire
PUFA: Polyunsaturated fatty acid
RA: Rheumatoid arthritis
rMT: Resting motor threshold
ROM: Range of motion
r_s_: Spearman correlation coefficient
Rv: Resolvin
SE: Standard error of the mean
SF: Synovial fluid
SFA: Saturated fatty acid
TKA: Total knee arthroplasty
TNF-*α*: Tumor necrosis factor-*α*
TPD: Two-point discrimination
VAS: Visual analog scale
WOMAC: Western Ontario and McMaster Universities Osteoarthritis Index
