# Supplementary material for: Plasma fatty acids reflect pain, disability, and psychological well-being in knee osteoarthritis in a longitudinal study with joint replacement surgery
Source: Sci Rep. 2026 Jan 22;16:6022. doi: 10.1038/s41598-026-36812-8 (PMC12902111; doi:10.1038/s41598-026-36812-8)
Supplement: Supplementary file 1 — Supplementary Material 1 [file 41598_2026_36812_MOESM1_ESM.pdf]

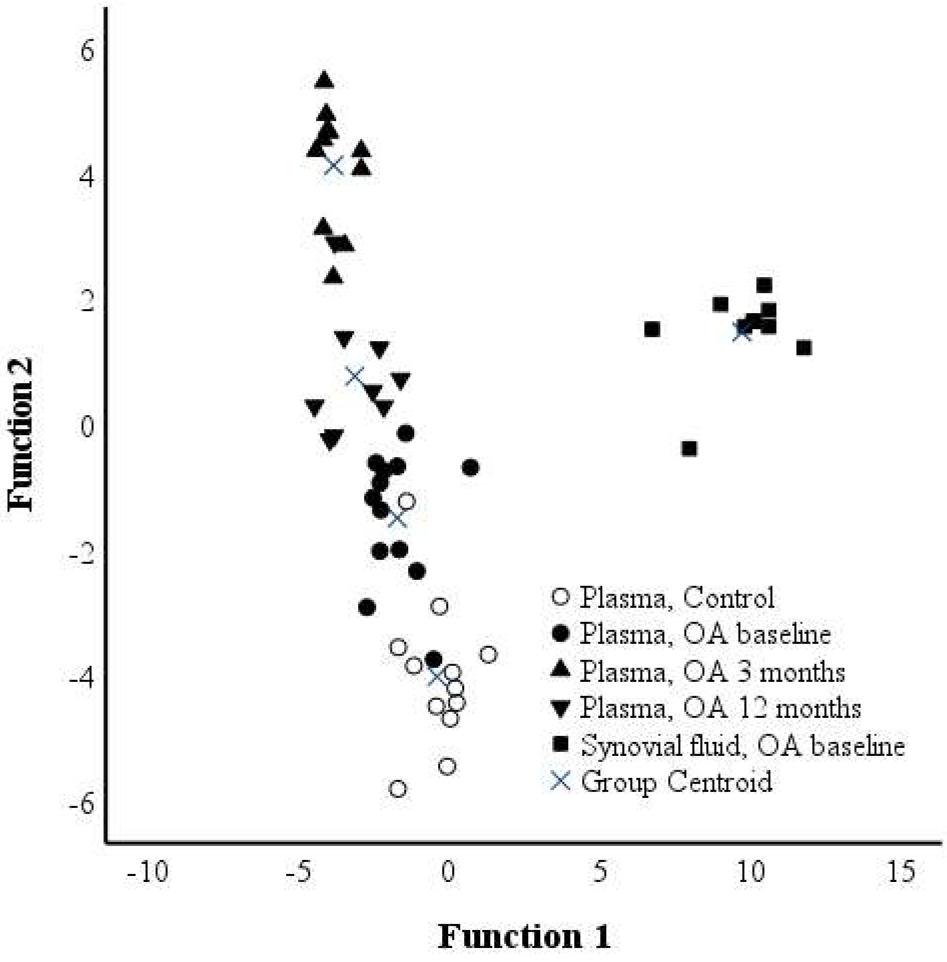

Supplementary Figure S1. Discriminant analysis depicting the classification of plasma and synovial fluid fatty acid data (mol-%) in controls and osteoarthritis (OA) patients. The discriminant functions 1 (on the x-axis) and 2 (y-axis) together explain 86.3% of the variance in the dataset. White symbols = controls, black symbols = OA patients.
