# Supplementary figures and images for: Plasma fatty acids reflect pain, disability, and psychological well-being in knee osteoarthritis in a longitudinal study with joint replacement surgery
Source: Sci Rep. 2026 Jan 22;16:6022. doi: 10.1038/s41598-026-36812-8 (PMC12902111; doi:10.1038/s41598-026-36812-8)

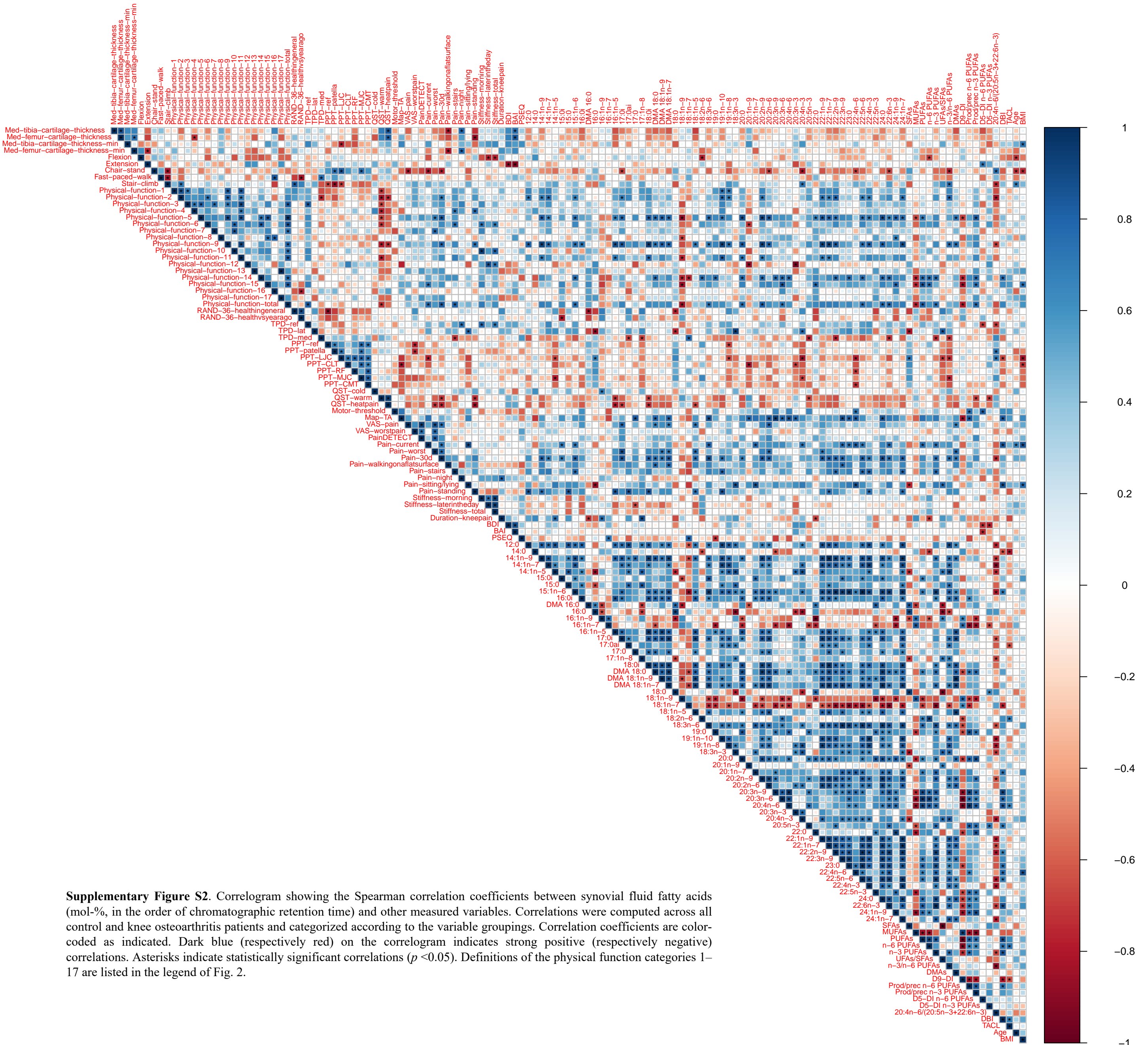

Supplement: Supplementary file 2 — Supplementary Material 2 [file 41598_2026_36812_MOESM2_ESM.pdf]
