# Supplementary material for: Plasma fatty acids reflect pain, disability, and psychological well-being in knee osteoarthritis in a longitudinal study with joint replacement surgery
Source: Sci Rep. 2026 Jan 22;16:6022. doi: 10.1038/s41598-026-36812-8 (PMC12902111; doi:10.1038/s41598-026-36812-8)
Supplement: Supplementary file 3 — Supplementary Material 3 [file 41598_2026_36812_MOESM3_ESM.pdf]

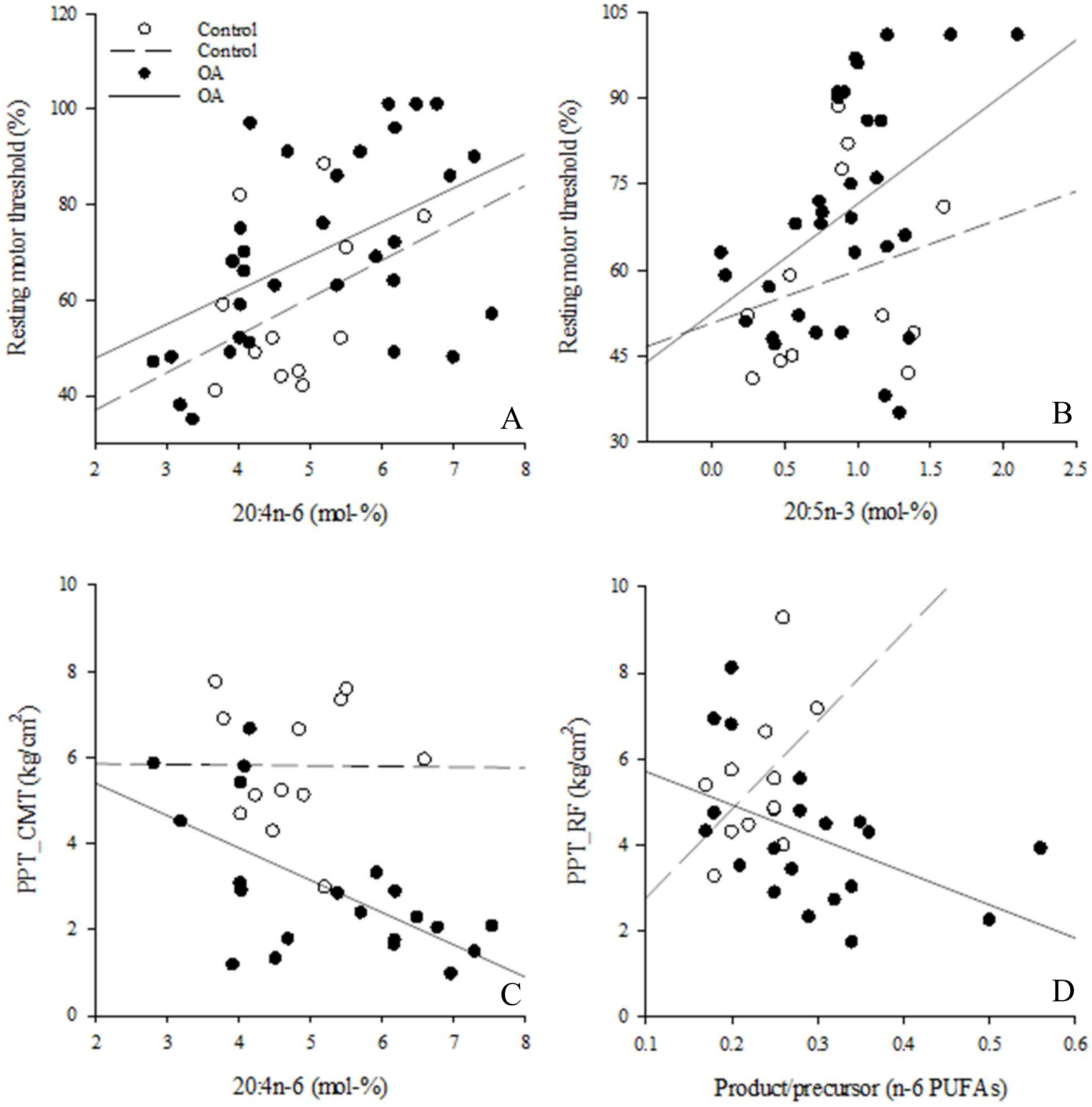

Supplementary Figure S3. Scatter plots depicting the interrelationships between selected plasma fatty acid variables and resting motor threshold (A–B) and pressure pain threshold (PPT; C–D) in controls and osteoarthritis (OA) patients. The R^2 and *p* values can be found in Table 2. CMT = medial tibial condyle, RF = *rectus femoris* muscle, PUFA = polyunsaturated fatty acid, white symbols/dashed line = controls, black symbols/solid line = OA patients.
