# Supplementary material for: Plasma fatty acids reflect pain, disability, and psychological well-being in knee osteoarthritis in a longitudinal study with joint replacement surgery
Source: Sci Rep. 2026 Jan 22;16:6022. doi: 10.1038/s41598-026-36812-8 (PMC12902111; doi:10.1038/s41598-026-36812-8)
Supplement: Supplementary file 4 — Supplementary Material 4 [file 41598_2026_36812_MOESM4_ESM.pdf]

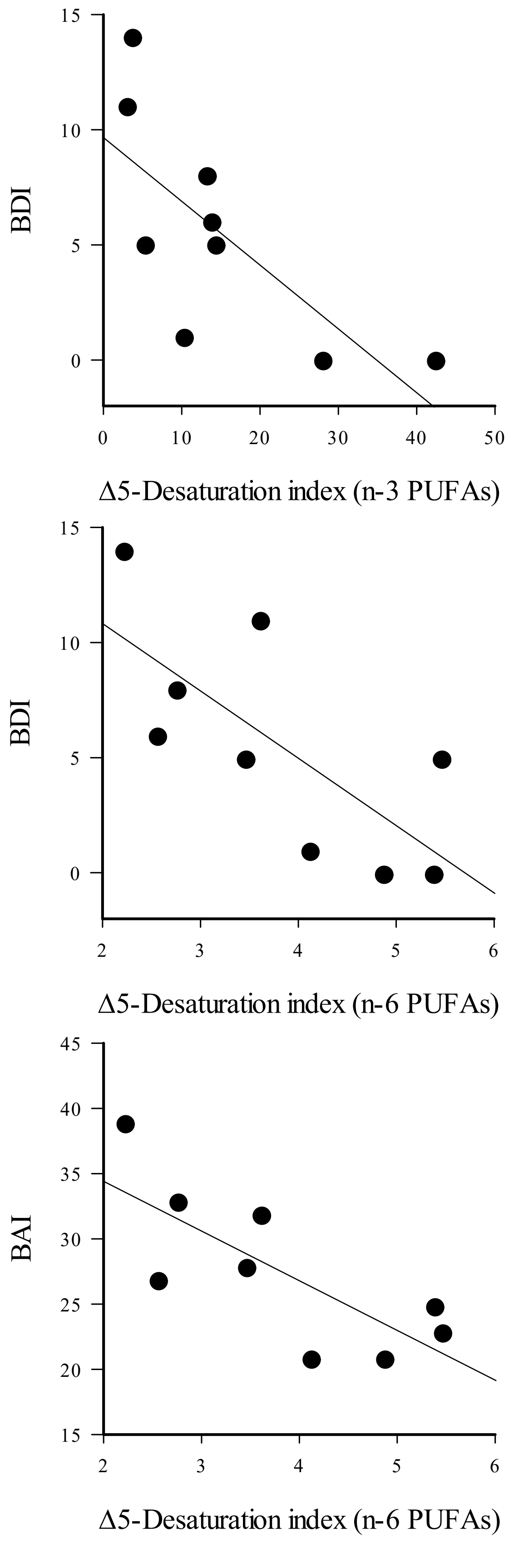

Supplementary Figure S4. Scatter plots depicting the interrelationships between synovial fluid Δ5-desaturation indices and Beck depression (BDI) or anxiety inventory (BAI) scores in knee osteoarthritis patients. The R^2 and *p* values can be found in Supplementary Table S2. PUFA = polyunsaturated fatty acid.
