# Supplementary material for: Plasma fatty acids reflect pain, disability, and psychological well-being in knee osteoarthritis in a longitudinal study with joint replacement surgery
Source: Sci Rep. 2026 Jan 22;16:6022. doi: 10.1038/s41598-026-36812-8 (PMC12902111; doi:10.1038/s41598-026-36812-8)
Supplement: Supplementary file 5 — Supplementary Material 5 [file 41598_2026_36812_MOESM5_ESM.pdf]

Supplementary Table S1. Proportions (mol-%) of fatty acids and alkenyl chains and their sums and ratios in the plasma (P) and synovial fluid (SF) of controls and osteoarthritis (OA) patients (mean ± SE, *n* = 9–13/group).

Fatty acid	Control P, baseline	OA P, baseline	OA P, 3 months	OA P, 12 months	p^{a}	OA SF, baseline
12:0	0.306 ± 0.074	0.327 ± 0.066	0.345 ± 0.071	0.321 ± 0.069	0.737	1.054 ± 0.308
14:0	1.718 ± 0.158	1.810 ± 0.191	1.860 ± 0.171	2.071 ± 0.235	0.579	2.961 ± 0.344
14:1n-9	0.045 ± 0.014	0.043 ± 0.011	0.075 ± 0.023	0.041 ± 0.011	0.120	0.238 ± 0.130
14:1n-7	0.072 ± 0.031	0.087 ± 0.026	0.083 ± 0.028	0.068 ± 0.023	0.945	0.551 ± 0.253
14:1n-5	0.205 ± 0.043	0.271 ± 0.057	0.199 ± 0.042	0.205 ± 0.036	0.434	0.829 ± 0.178
15:0*i*	0.072 ± 0.012	0.280 ± 0.117	0.254 ± 0.146	0.073 ± 0.026	0.076	0.151 ± 0.055
15:0	0.305 ± 0.022	0.266 ± 0.019	0.259 ± 0.024	0.293 ± 0.033	0.557	0.446 ± 0.106
15:1n-6	0.287 ± 0.148	0.303 ± 0.099	0.189 ± 0.086	0.178 ± 0.062	0.772	1.484 ± 0.653
16:0*i*	0.125 ± 0.026	0.125 ± 0.018	0.138 ± 0.034	0.126 ± 0.032	0.781	0.257 ± 0.090
DMA 16:0	0.334 ± 0.030	0.321 ± 0.035	0.337 ± 0.036	0.319 ± 0.047	0.927	0.359 ± 0.048
16:0	23.974 ± 0.752	24.534 ± 0.614	24.340 ± 0.671	24.924 ± 0.740	0.412	22.106 ± 1.583
16:1n-9	0.392 ± 0.046	0.421 ± 0.031	0.366 ± 0.030	0.443 ± 0.058	0.589	0.447 ± 0.045
16:1n-7	2.347 ± 0.256	3.698 ± 0.345	2.905 ± 0.312	3.408 ± 0.435	0.016	5.527 ± 0.737
16:1n-5	0.211 ± 0.040	0.159 ± 0.014	0.239 ± 0.043	0.163 ± 0.026	0.333	0.394 ± 0.077
17:0*i*	0.044 ± 0.009	0.034 ± 0.004	0.065 ± 0.019	0.036 ± 0.005	0.047	0.109 ± 0.029
17:0*ai*	0.171 ± 0.012	0.158 ± 0.008	0.183 ± 0.025	0.159 ± 0.007	0.257	0.367 ± 0.078
17:0	0.328 ± 0.073	0.185 ± 0.018	0.271 ± 0.059	0.213 ± 0.025	0.160	0.269 ± 0.060
17:1n-8	0.175 ± 0.038	0.150 ± 0.014	0.163 ± 0.019	0.181 ± 0.013	0.829	0.423 ± 0.122
18:0*i*	0.111 ± 0.025	0.088 ± 0.023	0.128 ± 0.027	0.086 ± 0.021	0.453	0.363 ± 0.083
DMA 18:0	0.178 ± 0.017	0.189 ± 0.023	0.196 ± 0.019	0.159 ± 0.037	0.793	0.211 ± 0.033
DMA 18:1n-9	0.228 ± 0.057	0.163 ± 0.027	0.268 ± 0.060	0.172 ± 0.054	0.477	0.640 ± 0.166
DMA 18:1n-7	0.105 ± 0.022	0.114 ± 0.032	0.095 ± 0.014	0.069 ± 0.015	0.528	0.270 ± 0.074
18:0	6.951 ± 0.791	6.103 ± 0.460	7.272 ± 0.655	6.348 ± 0.795	0.595	5.940 ± 1.008
18:1n-9	20.957 ± 0.779	21.189 ± 0.571	22.466 ± 0.909	22.547 ± 1.118	0.338	27.184 ± 2.832
18:1n-7	1.752 ± 0.085	1.943 ± 0.126	1.847 ± 0.079	1.974 ± 0.190	0.738	2.212 ± 0.219
18:1n-5	0.060 ± 0.013	0.062 ± 0.010	0.065 ± 0.011	0.054 ± 0.007	0.896	0.177 ± 0.047
18:2n-6	25.832 ± 1.300	23.080 ± 1.199	22.106 ± 0.908	23.206 ± 1.599	0.209	13.356 ± 1.143
18:3n-6	0.276 ± 0.056	0.419 ± 0.064	0.372 ± 0.067	0.348 ± 0.094	0.478	0.292 ± 0.108
18:3n-3	0.941 ± 0.119	0.862 ± 0.057	0.833 ± 0.063	0.888 ± 0.099	0.919	0.916 ± 0.144
19:0	0.301 ± 0.189	0.095 ± 0.049	0.081 ± 0.017	0.111 ± 0.059	0.306	0.208 ± 0.051
19:1n-10	0.098 ± 0.029	0.064 ± 0.010	0.137 ± 0.057	0.062 ± 0.013	0.114	0.288 ± 0.091
19:1n-8	0.076 ± 0.015	0.071 ± 0.007	0.076 ± 0.014	0.085 ± 0.010	0.957	0.140 ± 0.037
20:0	0.151 ± 0.019	0.187 ± 0.021	0.156 ± 0.012	0.148 ± 0.017	0.424	0.201 ± 0.049
20:1n-9	0.172 ± 0.031	0.152 ± 0.022	0.176 ± 0.028	0.143 ± 0.018	0.822	0.270 ± 0.038
20:1n-7	0.033 ± 0.012	0.041 ± 0.010	0.038 ± 0.009	0.038 ± 0.012	0.899	0.099 ± 0.036
20:2n-9	0.041 ± 0.012	0.049 ± 0.015	0.049 ± 0.017	0.033 ± 0.004	0.446	0.335 ± 0.191
20:2n-6	0.171 ± 0.031	0.160 ± 0.023	0.158 ± 0.017	0.145 ± 0.010	0.892	0.244 ± 0.041
20:3n-9	0.105 ± 0.019	0.130 ± 0.015	0.135 ± 0.016	0.155 ± 0.020	0.297	0.168 ± 0.075
20:3n-6	1.113 ± 0.051	1.347 ± 0.084	1.254 ± 0.051	1.189 ± 0.073	0.103	0.869 ± 0.172
20:3n-3	0.055 ± 0.023	0.054 ± 0.025	0.104 ± 0.049	0.025 ± 0.006	0.310	0.080 ± 0.025
20:4n-6	4.775 ± 0.241	5.198 ± 0.383	4.760 ± 0.388	5.195 ± 0.491	0.913	3.103 ± 0.536
20:4n-3	0.059 ± 0.011	0.075 ± 0.014	0.061 ± 0.012	0.071 ± 0.011	0.777	0.054 ± 0.012
20:5n-3	0.860 ± 0.130	0.984 ± 0.118	0.934 ± 0.124	0.748 ± 0.150	0.596	0.559 ± 0.135
22:0	0.268 ± 0.046	0.389 ± 0.058	0.324 ± 0.039	0.219 ± 0.030	0.061	0.215 ± 0.057
22:1n-9	0.053 ± 0.017	0.053 ± 0.026	0.044 ± 0.019	0.025 ± 0.004	0.672	0.047 ± 0.011
22:1n-7	0.018 ± 0.009	0.019 ± 0.006	0.026 ± 0.011	0.017 ± 0.004	0.423	0.041 ± 0.013
22:2n-9	0.011 ± 0.003	0.016 ± 0.004	0.020 ± 0.007	0.014 ± 0.004	0.110	0.046 ± 0.017
22:3n-9	0.080 ± 0.020	0.080 ± 0.028	0.132 ± 0.090	0.020 ± 0.005	0.124	0.296 ± 0.165
22:4n-6	0.080 ± 0.008	0.101 ± 0.009	0.079 ± 0.010	0.093 ± 0.030	0.607	0.168 ± 0.026
22:4n-3	0.015 ± 0.004	0.020 ± 0.005	0.013 ± 0.002	0.011 ± 0.003	0.097	0.053 ± 0.023
22:5n-6	0.030 ± 0.009	0.036 ± 0.007	0.034 ± 0.006	0.025 ± 0.005	0.413	0.081 ± 0.031
22:5n-3	0.447 ± 0.029	0.438 ± 0.024	0.467 ± 0.063	0.342 ± 0.048	0.034	0.463 ± 0.068
22:6n-3	1.754 ± 0.120	1.695 ± 0.117	2.015 ± 0.422	1.417 ± 0.185	0.020	1.530 ± 0.445
23:0	0.081 ± 0.016	0.136 ± 0.025	0.076 ± 0.013	0.065 ± 0.011	0.078	0.175 ± 0.072
24:0	0.211 ± 0.032	0.336 ± 0.056	0.267 ± 0.025	0.183 ± 0.024	0.042	0.311 ± 0.096
24:1n-9	0.415 ± 0.068	0.653 ± 0.095	0.420 ± 0.058	0.322 ± 0.057	0.033	0.343 ± 0.075
24:1n-7	0.024 ± 0.006	0.032 ± 0.006	0.045 ± 0.017	0.024 ± 0.006	0.054	0.074 ± 0.032
∑:SFA	35.118 ± 1.374	35.053 ± 0.772	36.021 ± 0.921	35.375 ± 1.123	0.973	35.134 ± 1.969
∑:MUFA	27.392 ± 0.653	29.412 ± 0.838	29.557 ± 0.570	29.979 ± 1.601	0.227	40.770 ± 3.046
∑:PUFA	36.644 ± 1.749	34.748 ± 1.181	33.527 ± 1.328	33.926 ± 1.791	0.499	22.615 ± 2.379
∑:n-6 PUFA	32.277 ± 1.486	30.343 ± 1.202	28.763 ± 0.938	30.202 ± 1.659	0.323	18.113 ± 1.702
∑:n-3 PUFA	4.131 ± 0.367	4.129 ± 0.204	4.427 ± 0.602	3.502 ± 0.393	0.108	3.656 ± 0.758
n-3/n-6 PUFA	0.127 ± 0.009	0.140 ± 0.010	0.153 ± 0.018	0.117 ± 0.012	0.036	0.199 ± 0.031
∑:DMA	0.845 ± 0.089	0.787 ± 0.090	0.896 ± 0.101	0.720 ± 0.147	0.651	1.480 ± 0.272

UFA/SFA	1.871 ± 0.111	1.846 ± 0.059	1.769 ± 0.071	1.830 ± 0.095	0.906	1.890 ± 0.172
Delta-9-DI	0.776 ± 0.055	0.868 ± 0.055	0.900 ± 0.048	0.917 ± 0.087	0.412	2.170 ± 0.454
Delta-6-DI (n-6)	0.011 ± 0.002	0.020 ± 0.004	0.017 ± 0.003	0.015 ± 0.003	0.283	0.022 ± 0.009
Delta-5-DI (n-6)	4.325 ± 0.176	4.016 ± 0.388	3.866 ± 0.361	4.673 ± 0.715	0.584	3.779 ± 0.365
Delta-5-DI (n-3)	19.784 ± 4.274	24.303 ± 8.662	24.843 ± 6.472	15.807 ± 4.939	0.810	13.963 ± 3.954
Prod/prec (n-6)	0.231 ± 0.011	0.296 ± 0.028	0.279 ± 0.025	0.287 ± 0.032	0.394	0.289 ± 0.043
Prod/prec (n-3)	2.956 ± 0.239	3.235 ± 0.248	3.449 ± 0.430	2.617 ± 0.417	0.141	2.275 ± 0.424
20:4n-6/(20:5n-3+22:6n-3)	1.968 ± 0.158	2.013 ± 0.146	2.948 ± 1.288	3.068 ± 0.765	0.520	1.935 ± 0.300
DBI	1.233 ± 0.043	1.225 ± 0.024	1.205 ± 0.040	1.192 ± 0.036	0.740	1.039 ± 0.064
TACL	17.489 ± 0.037	17.493 ± 0.029	17.475 ± 0.044	17.435 ± 0.027	0.597	17.134 ± 0.065

i = *iso*-methyl-branch, *ai* = *anteiso*-methyl-branch, DMA = dimethyl acetal (derivative of alkenyl chain), SFA = saturated fatty acid, MUFA = monounsaturated fatty acid, PUFA = polyunsaturated fatty acid, UFA = unsaturated fatty acid (MUFA + PUFA), DI = desaturation index, prod = product, prec = precursor, DBI = double bond index, TACL = total average chain length, [a]the effect of group in the generalized linear model within plasma samples
