# Supplementary material for: Plasma fatty acids reflect pain, disability, and psychological well-being in knee osteoarthritis in a longitudinal study with joint replacement surgery
Source: Sci Rep. 2026 Jan 22;16:6022. doi: 10.1038/s41598-026-36812-8 (PMC12902111; doi:10.1038/s41598-026-36812-8)
Supplement: Supplementary file 6 — Supplementary Material 6 [file 41598_2026_36812_MOESM6_ESM.pdf]

Supplementary Table S2. Univariate analyses of variance for physiatric measurements explained by different fatty acid variables in the synovial fluid of knee osteoarthritis patients. Only analyses that are statistically significant when adjusted for age and body mass index are reported.

Fatty acid	Dependent variable	R squared	F	*P*
16:0	Two-point discrimination, lateral	0.766	10.428	0.023
	Pain, sitting or lying down	0.903	12.988	0.023
16:1n-7	Pain self-efficacy	0.724	8.641	0.042
18:3n-6	Pain, current	0.822	8.515	0.033
18:3n-3	Two-point discrimination, lateral	0.885	26.322	0.004
	Pain, sitting or lying down	0.939	23.115	0.009
22:6n-3	Pain, current	0.793	6.629	0.050
PUFA	Pain, current	0.833	9.387	0.028
DMA	VAS pain	0.664	7.739	0.039
	Pain, 30d	0.830	17.089	0.009
DBI	Pain, current	0.830	9.116	0.029
	Pain, sitting or lying down	0.882	9.948	0.034
Delta5-DI (n-6)	Two-point discrimination, reference	0.732	9.156	0.029
	Beck depression inventory	0.597	6.959	0.046
	Beck anxiety inventory	0.709	9.729	0.026
Delta5-DI (n-3)	Beck depression inventory	0.671	9.658	0.027

PUFA = polyunsaturated fatty acid, DMA = dimethyl acetal (derivative of alkenyl chain), DBI = double bond index, Delta5-DI (n-6) = 20:4n-6/20:3n-6, Delta5-DI (n-3) = 20:5n-3/20:4n-3, VAS = visual analog scale
